# Microanatomical findings with relevance to trigeminal ganglion enhancement on post-contrast T1-weighted magnetic resonance images in dogs

**DOI:** 10.3389/fvets.2023.1256947

**Published:** 2023-09-13

**Authors:** Koen M. Santifort, Eric N. Glass, Marti Pumarola, Vicente Aige Gil

**Affiliations:** ^1^IVC Evidensia Small Animal Referral Hospital Arnhem, Neurology, Arnhem, Netherlands; ^2^IVC Evidensia Small Animal Referral Hospital Hart van Brabant, Neurology, Waalwijk, Netherlands; ^3^Section of Neurology and Neurosurgery, Red Bank Veterinary Hospital, Tinton Falls, NJ, United States; ^4^Unit of Compared and Murine Pathology, Department of Animal Medicine and Surgery, Faculty of Veterinary Medicine, Universitat Autònoma de Barcelona, Barcelona, Spain; ^5^Department of Sanitat i Anatomía Animal, Faculty of Veterinary Medicine, Universitat Autònoma de Barcelona, Barcelona, Spain

**Keywords:** trigeminal ganglion, gadolinium, blood-nerve barrier, blood-ganglion barrier, contrast enhancement

## Abstract

**Introduction:**

Trigeminal ganglion contrast enhancement (TGCE) is reported to be a normal and a common finding on magnetic resonance imaging studies of dogs, cats and humans. The intent of the present study was to describe the anatomical characteristics of the trigeminal ganglion, its surrounding structures, and histological features that are relevant to explain or hypothesize on the reason for TGCE on T1-weighted post-contrast MRI studies of the brain in dogs.

**Methods:**

Eight dog cadavers were dissected to study the anatomy of the trigeminal ganglion. The presence and anatomy of vessels was studied by dissection and by histological techniques. Two trigeminal ganglia were isolated and stained with hematoxylin–eosin (HE). Two other trigeminal ganglia included in the trigeminal canal and trigeminal cavity were decalcified with formic acid/formalin for 12 weeks and stained with HE to study the related vessels. Additionally, a corrosion cast was obtained from a separate canine specimen.

**Results:**

Leptomeninges and a subarachnoid space were identified at the level of the trigeminal nerve roots and the trigeminal ganglion. No subarachnoid space was identified and leptomeninges were no longer present at the level of the three trigeminal nerve branches. Small arterial vessels ran to and supplied the trigeminal ganglion, passing through the dura mater. No venous plexus was visualized at the level of the trigeminal ganglion in the dissections. A complex arterial vascular network was identified within the leptomeningeal covering of the trigeminal ganglion and was best appreciated in the corrosion cast. Histological examination revealed small-to moderate-sized blood vessels located in the epineurium around the ganglion; from there a multitude of arterioles penetrated into the perineurium. Small endoneurial branches and capillaries penetrated the ganglion and the trigeminal nerve branches.

**Discussion:**

Limitations to this study include the limited number of canine specimens included and the lack of electron microscopy to further support current hypotheses included in our discussion. In conclusion, this study provides further support to the theory that TGCE in dogs may be due an incomplete blood-nerve barrier or blood-ganglion barrier at the interface between the central nervous system and the peripheral nervous system.

## Introduction

**Figure 1:**
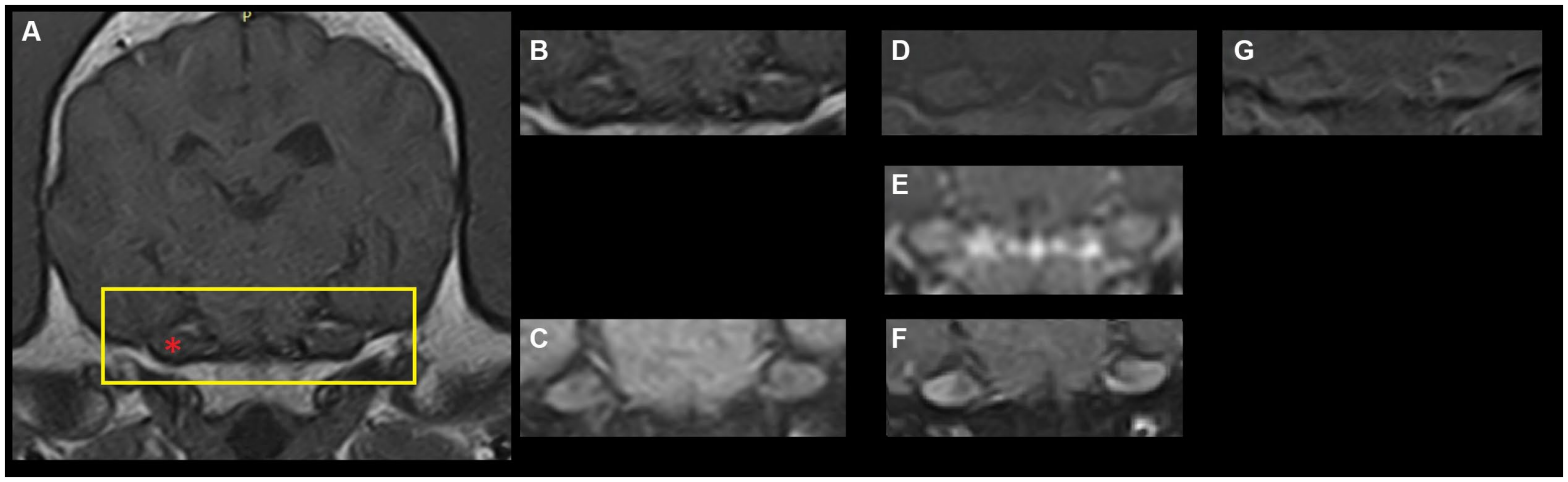
Archival magnetic resonance images (1.5 T-Canon Vantage Elan, The Netherlands) of a dog’s brain at the level of the trigeminal ganglion. **(A)** T1-weighted fast-spin echo pre-contrast. The yellow rectangle depicts the region shown in panels **(B–G)**. The red asterisk point out the right trigeminal ganglion (left of the image). **(B)** T1-weighted fast-spin echo pre-contrast. **(C)** T1-weighted fast-spin echo with fat saturation pre-contrast. **(D)** T1-weighted fast-spin echo post-contrast. **(E)** Three-dimensional T1-weighted gradient echo post-contrast. **(F)** T1-weighted fast-spin echo with fat saturation post-contrast. **(G)** Subtraction image (T1-weighted post-contrast minus T1-weighted pre-contrast).

Magnetic resonance imaging (MRI) studies of the canine and feline head offers clinicians the opportunity to diagnose a plethora of disorders affecting the brain, cranial nerves and surrounding structures. Publications describing normal findings, so-called pseudolesions, and anatomical features regarding, for instance, cranial nerves exiting from the cranium provide a valuable resource for comparison to pathological states (1–5). A number of these studies highlight that the canine and feline trigeminal ganglion, ophthalmic, maxillary and mandibular nerves and/or trigeminal nerve roots show T1-weighted (T1W) post-contrast (gadolinium) enhancement on MRI studies in non-pathological states (1–3, 6, 7). Briefly, the presence of gadolinium in anatomical structures results in increased signal intensity on T1W images (i.e., structures become more white on post-contrast images compared to pre-contrast images). This finding has been reported in 93–100% of normal dogs (2, 6, 7). Thus, trigeminal ganglion contrast enhancement (TGCE) is regarded as a normal finding in dogs (Figure 1).

It is valuable for clinicians to be aware of this normal finding, as it may be mistaken for an abnormality (pseudolesion), and it be mistakenly thought of as a pathologic process affecting the trigeminal ganglion (1). As for other intracranial structures that show contrast enhancement in non-pathological or normal states, there should be an anatomical explanation for this finding of enhancement of trigeminal structures. For example, dynamic MRI studies showed that normal contrast-enhancement of the canine hypophysis is related to its (micro)circulatory anatomy (8).

In human literature, two hypotheses are put forward to explain normal TGCE. These have been based a few articles in particular. One hypothesis is that vessels supplying the trigeminal ganglion are permeable, thus accounting for gadolinium leakage and presence in trigeminal ganglia and resulting in TGCE. This is largely based on human studies regarding specific sections of multiple (branches of) cranial nerves and MRI observations (9, 10). Such vascular permeability suggests that the blood-nerve barrier (BNB) or blood-ganglion barrier (BGB) at that level is incomplete. Therefore, an incomplete trigeminal BGB may account for TGCE. The other hypothesis is based on the presence of perineural/periganglionic vascular networks that are proposed to cause the appearance of TGCE on MRI in humans (11). There are no published studies that report specific anatomical explanations for TGCE in dogs. Some authors have put forward a hypothesis, mostly based on studies in rabbits (1–3, 7, 12, 13). Researchers observed fluorescence in trigeminal structures after intravenous fluorescein administration, suggesting vascular permeability (12, 13). Authors of a veterinary publication discussing these hypotheses have noted that ‘*cadaveric studies in dogs would be useful to determine whether a perineural venous plexus is present, paralleling what has been documented in humans*’ (1). Although anatomical descriptions of local anatomy regarding the trigeminal ganglion in dogs exist (14), no studies specifically address microanatomical features with relevance to explanations for TGCE.

The objective of this study was to describe the anatomical characteristics of the trigeminal ganglion, its surrounding structures, and histological features that are relevant to explain or hypothesize the cause for TGCE on T1-weighted post-contrast MRI studies of the brain in dogs. In the discussion, we include a brief literature review regarding TGCE and hypothesize on the reason for TGCE in dogs based on this review and our findings.

## Materials and methods

Eight adult dog cadavers were dissected to study the anatomy of the trigeminal ganglion. The cadavers used came from the dissection room of the Anatomy Unit of the Veterinary Faculty of the Universitat Autónoma de Barcelona. The dogs were euthanized for medical reasons unrelated to the central nervous system (CNS) and donated by owners following the approved donation program of the University and used for anatomical dissections. All the specimens were dissected by one author (VAG).

Dog breeds included:

- 3 beagles (females)
- 1 German shepherd (male)
- 1 golden retriever (male)
- 1 Border collie (female)
- 2 mixed breeds (female)

Cadavers were fixed with a 10% formaldehyde buffered solution injected via the common carotid artery. The cadavers were then preserved for a few weeks (variable) at 4–6°C. The heads of the eight dogs were isolated by atlantooccipital decapitation.

Dorsal craniotomies were performed on 5 heads (beagle, German shepherd, Border collie, 2 mixed breeds). The brains were removed by gross dissection.

The other heads (2 beagles, 1 golden retriever) were transversally sectioned.

For each, dissection of the trigeminal ganglion and related structures was performed from that point forward with the use of stereomicroscopy and microsurgical equipment.

The presence and anatomy of vessels was studied by dissection and by histological techniques. Two trigeminal ganglia were isolated and stained with hematoxylin–eosin (HE). Two other trigeminal ganglia included in the trigeminal canal and trigeminal cavity were decalcified with formic acid/formalin for 12 weeks and stained with HE to study the related vessels. Additionally, a corrosion cast was obtained from a separate, additional (ninth) canine specimen (beagle, male) injecting araldite mixed with red die (Monomer CY223, hardener HY2967–Huntsman Advanced Materials, USA) through the carotid artery. Briefly, the injected anatomical sample has been dissected and corroded in a basic solution of pancreatin at 37°C that destroys the muscles and ligaments while preserving the vascular mold.

## Results

### Trigeminal nerve roots, trigeminal ganglion and trigeminal nerves

**Figure 2:**
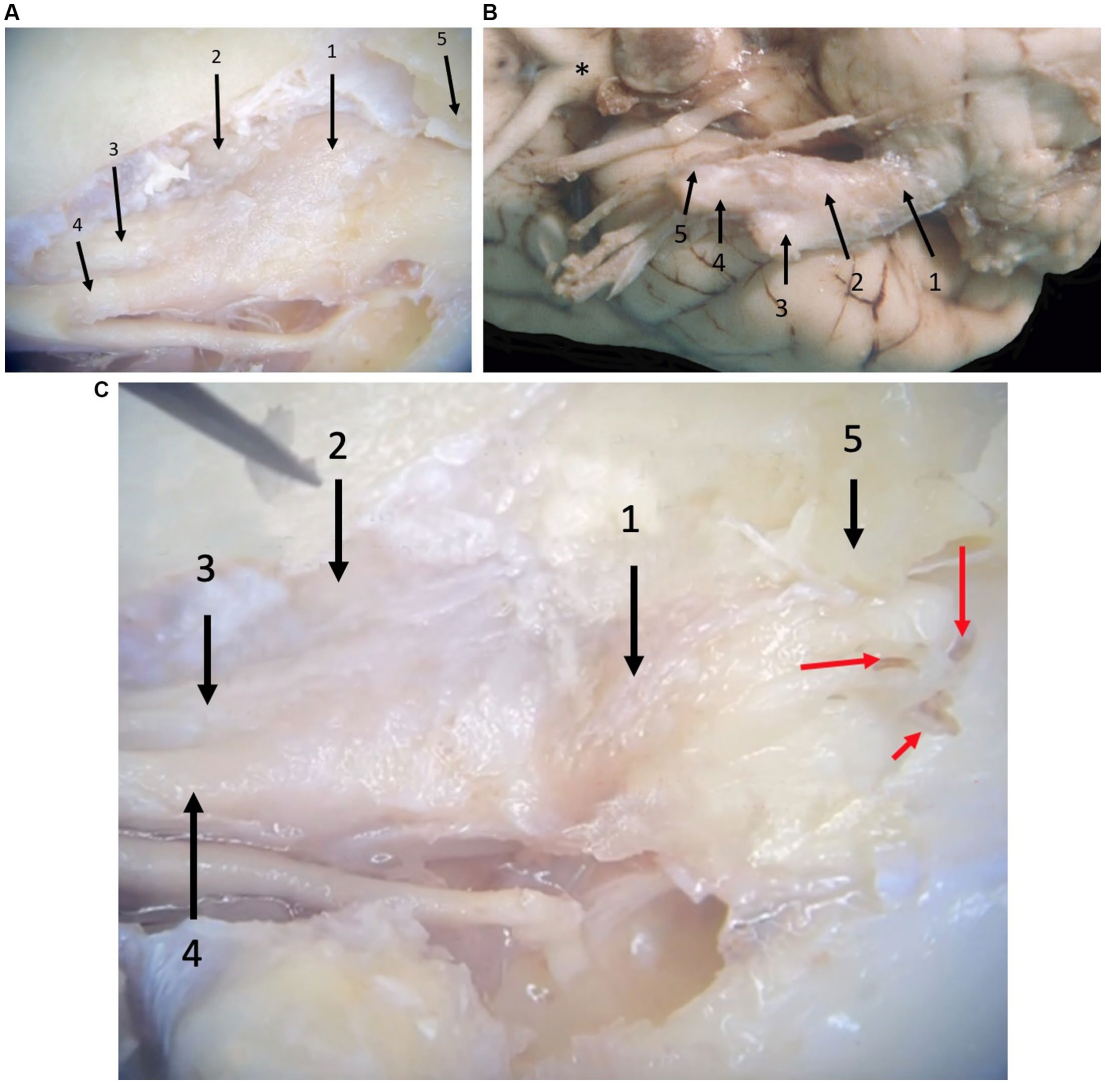
Photographs taken during microanatomical dissections. **(A)** Dorsal view of the right trigeminal ganglion surrounded by the leptomeninges inside the trigeminal cave. 1: trigeminal ganglion, 2: mandibular nerve, 3: maxillary nerve, 4: ophthalmic nerve, 5: crista petrosa. **(B)** Ventral view of the right trigeminal ganglion partially surrounded by the leptomeninges. 1: trigeminal nerve roots, 2: trigeminal ganglion, 3: mandibular nerve, 4: maxillary nerve, 5: ophthalmic nerve, *: optic chiasm. Rostral is left in the image. **(C)** Close-up of panel **(A)**, after removal of some meninges highlighting the presence of blood vessels (red arrows) that can be appreciated macroscopically in formalin-fixed dissection material. 1: trigeminal ganglion, 2: mandibular nerve, 3: maxillary nerve, 4: ophthalmic nerve, 5: trigeminal canal opened dorsally (petrosal crest removed).

From the ventrolateral aspect of the brain stem, trigeminal nerve roots course rostrally through the trigeminal canal formed by the petrosal crest (Figure 2). At is rostral extent, an osseous trigeminal cave (cavum trigeminale or ‘Merckel cave’–a term derived from human literature but not included in the Nomina Anatomica Veterinaria) is formed by a subtle depression of bone between the apex of the petrosal crest and the skull base, internally lined by a double layer of dura mater and the leptomeninges. The trigeminal ganglion is situated therein. From the ganglion rostrally, the trigeminal nerve branches into three nerves: the mandibular, the maxillary, and the ophthalmic nerves. These respective nerves are contained within the cranial cavity before leaving through their respective foramina/fissures: the oval foramen, round foramen and orbital fissure, respectively.

### Meninges

Leptomeninges and a subarachnoid space (trigeminal cistern–a term derived from human literature but not included in the Nomina Anatomica Veterinaria) were identified at the level of the trigeminal nerve roots and the trigeminal ganglion (Figure 2). Specifically, between the medulla oblongata and the caudal opening of the trigeminal canal, the trigeminal nerve roots are included in the subarachnoid space that contains arteries derived from the basilar artery as well as veins. At the level of the three trigeminal nerve branches (i.e., the mandibular, the maxillary, and the ophthalmic nerves), no subarachnoid space was identified and leptomeninges were no longer present.

### Vasculature

**Figure 3:**
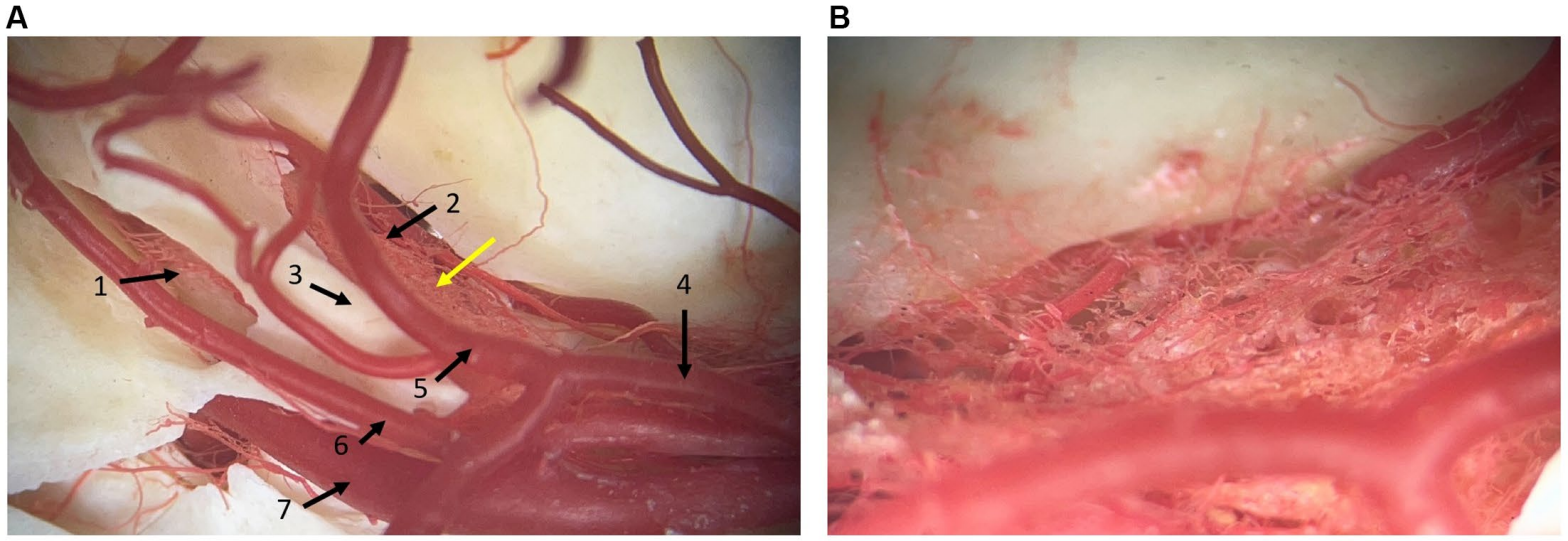
Photographs of a corrosion cast where arterial vessels are preserved by polymeric resin araldite and red dye. **(A)** dorsal view at the level of the junction between the middle and caudal cranial fossa. 1: caudal opening of the trigeminal canal, 2: rostral opening of the trigeminal canal, 3: crista petrosa, 4: caudal communicating artery of the circulus arteriosus cerebri, 5: caudal cerebral artery, 6: rostral cerebellar artery, 7: internal carotid artery. **(B)** Close-up at the level of the yellow arrow in panel **(A)**. It shows the vascular network located at the site of the trigeminal ganglion. Rostral is right in the image.

Small arterial vessels, coming from the basilar artery, ran to and supplied the trigeminal ganglion, passing through the dura mater (Figure 3). Furthermore, blood vessels that run with the trigeminal nerve and trigeminal nerve roots were identified. Venous plexuses were found at the level of the round and oval foramen near the maxillary and mandibular branches of the trigeminal nerve, respectively. No venous plexus was visualized at the level of the trigeminal ganglion in the dissections. A complex arterial vascular network was identified within the leptomeningeal covering of the trigeminal ganglion and was best appreciated in the corrosion cast (Figure 3).

**Figure 4:**
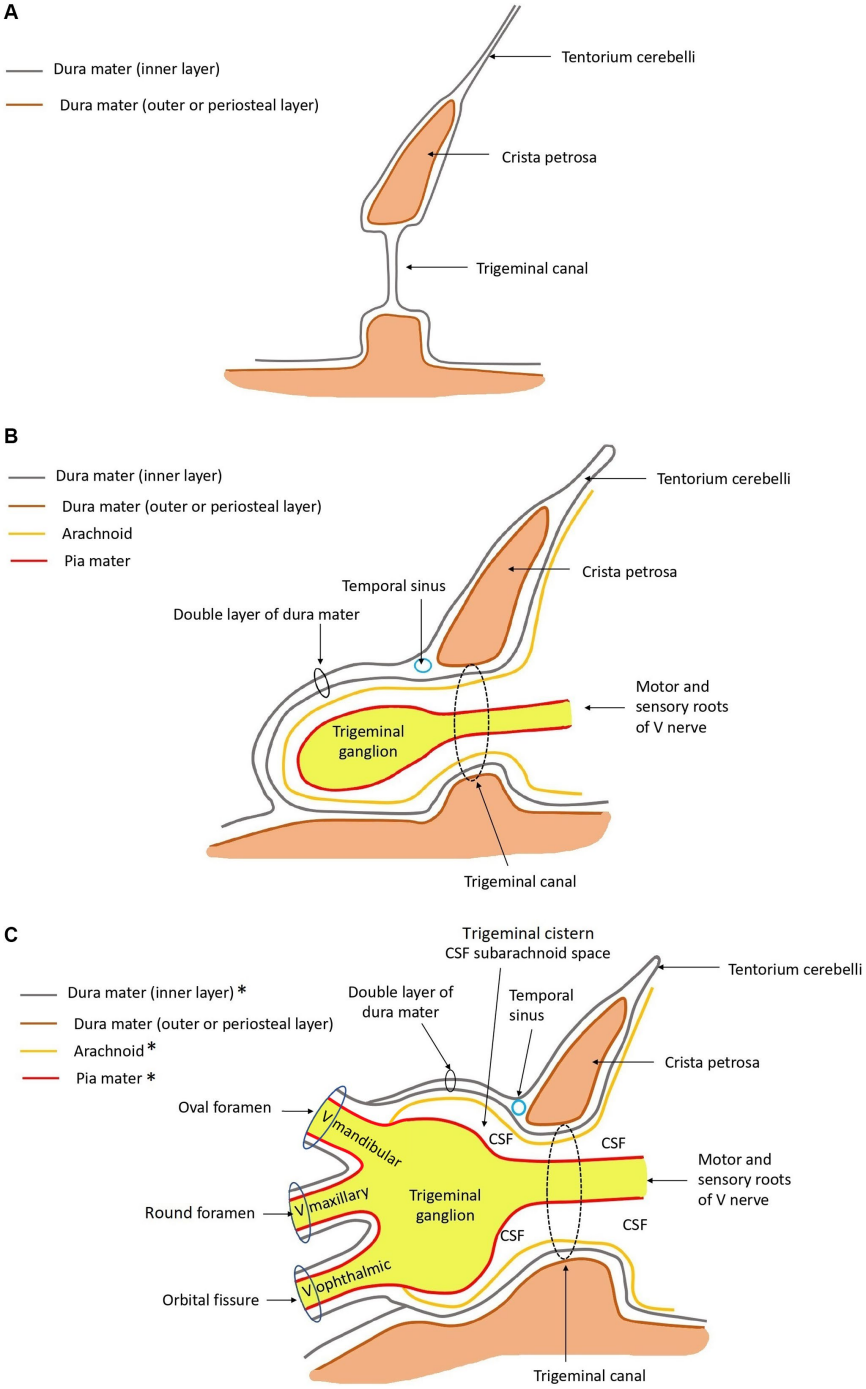
Schematic drawings in sagittal plane of the anatomy at the level of the trigeminal canal, trigeminal cave, and ophthalmic, maxillary, and mandibular nerves rostrally. *The ganglion and roots of the trigeminal nerve are covered by arachnoid and dura mater. The mandibular, maxillary, and ophthalmic nerves are not. These are covered by epineurium and perineurium, and contain endoneurium. Rostral is left in the images. **(A)** Only bone and dura mater are depicted. **(B)** Leptomeninges, roots of the trigeminal nerve, and the trigeminal ganglion are drawn in, in addition to panel **(A)**. **(C)** The mandibular, maxillary, and ophthalmic nerves are drawn in, in addition to panel **(B)**. The relationship with regard to the meningeal coverings are depicted.

Figure 4 depicts, schematically, the anatomical arrangement of the bony, meningeal and trigeminal structures.

**Figure 5:**
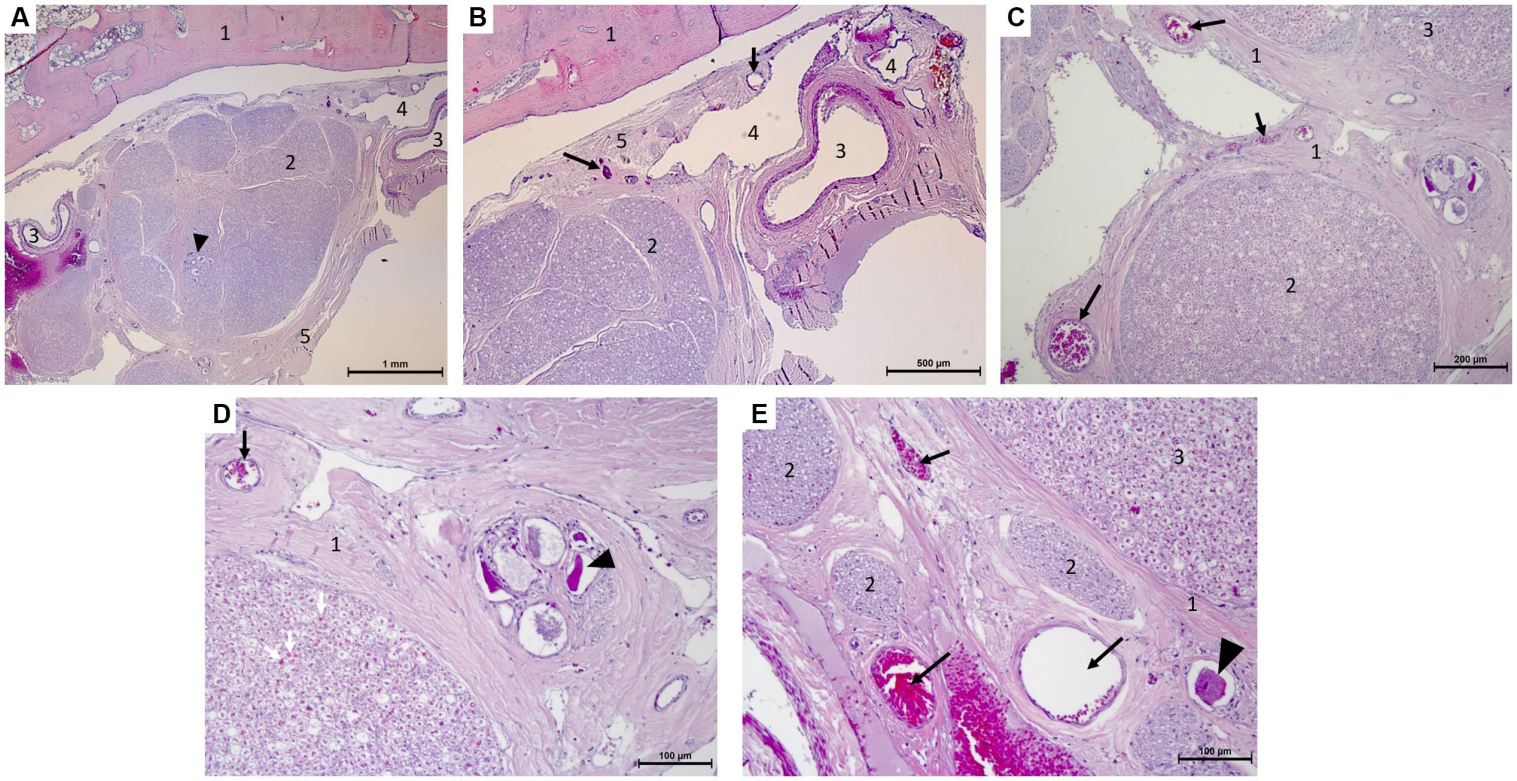
Microscopy photographs taken at the edge of the trigeminal ganglion histology (hematoxylin–eosin stain after decalcification with formic acid/formalin for 12 weeks). **(A,B)** [**(B)**:close-up of right top corner of panel **(A)**]: Panoramic view of the ganglion and bone with the vascular plexus on the right of the image. 1: temporal bone, 2: trigeminal ganglion with neuronal bodies (arrow head), 3: artery, 4: vein, 5: periosteal layer of the dura mater with capillaries (arrows). **(C,D)** [**(D)**:close-up of right half of panel **(C)**]: Capillaries in epi-, peri-, and endoneurium (black and white arrows) and capillaries surrounding a group of ganglionic neuronal bodies (arrow head) and collections of myelinated nerve fibers. 1: epineurium and perineurium with arterioles (arrows), 2: myelinated nerve fibers of a trigeminal nerve with capillaries in endoneurium (white arrows), 3: edge of the trigeminal ganglion. **(E)** Arterioles and capillaries surrounding an isolated ganglionic neuronal cell body (arrow head) and collections of myelinated nerve fibers. 1: epineurium and perineurium with capillaries (arrows), 2: myelinated nerve fibers of a trigeminal nerve, 3: edge of the trigeminal ganglion.

Histological examination revealed small-to moderate-sized blood vessels located in the epineurium around the ganglion (Figure 5). From there a multitude of arterioles emerged penetrating into the perineurium; the venous components were organized in large endothelium-lined cavities associated with the vascular plexuses adjacent to the trigeminal ganglion. Finally, small branches and capillaries penetrated the ganglion and the trigeminal nerve branches. These were distributed in the endoneurium (Figure 5).

## Discussion

In this canine cadaveric and histological study, we describe the presence of complex vascular networks around the trigeminal ganglion in dog specimens. When contrast medium (commonly gadolinium-based) is administered to canine patients undergoing MRI studies of the brain, the presence of vascular structures must be taken into account when assessing TGCE. In theory, enhancement of vascular structures might be mistaken for TGCE, especially when contrast- and spatial resolution are suboptimal. Indeed, a human cadaveric study described the presence of a perineural/periganglionic vascular plexus and postulated that contrast enhancement is the reason for apparent TGCE (11). The authors reported ‘true’ TGCE in only 4% of human trigeminal ganglia in that study. This study by Williams et al. is referenced frequently in the human literature when discussing TGCE. The results thereof contrast the study by Downs et al. who reported clear TGCE in 88% of cases and regional enhancement blending with the dura mater at this site in the remaining 12% (10). These authors postulated that TGCE is likely due to the presence of an incomplete BNB (or BGB) characterized by fenestrated capillaries at the level of the trigeminal ganglion as in spinal dorsal root ganglia (DRG) (10). Our microanatomical and histological findings of a complex periganglionic arterial vascular network covering and penetrating the trigeminal ganglion support the latter hypothesis.

Publications from human studies have, without additional evidence, taken the results of either or both of these studies (10, 11) into account when discussing TGCE. One review by Yousry et al. particularly addresses the differences between these studies and their hypotheses (15). Yousry et al. argued in favor of true TGCE, particularly evident using contrast enhanced three-dimensional (3D) constructive interference in steady state (CISS) sequences.

Our findings of a rich and complex vascular supply to the trigeminal ganglion contrast the findings in the human cadaveric study reported by Williams et al. (11). In that study, the trigeminal ganglion and proximal divisions of the trigeminal nerve were reported to be devoid of ‘obvious vascularity’. In the authors opinion, such findings are unlikely to reflect the anatomy of the dog and have indeed been proven to be inaccurate by other human (16) as well as canine (17) studies. Therefore, visual absence of vessels in the study by Williams et al. (11) must have been due to limitations of the study methodology. The trigeminal ganglion is populated by neuronal cell bodies. Neurons are among the most energy-consuming cell populations in the mammalian body and vascularization is essential for provision of substances and clearing of waste products in such a metabolically active structure (16). Supplementary Figure 1 illustrates the general anatomy of the vasa nervorum and their derivatives. Our own observations in this canine study of a rich and complex vascular blood supply, support that this general anatomy is applicable to the trigeminal nerve roots, trigeminal nerves, and trigeminal ganglion as well.

The presence of tight junctions and non-fenestrated endothelium characterizes the BNB (18–21). But this BNB is not necessarily identical at every location (22). The term ‘BNB’ is often applied to the ganglion of the nerve in question as well. However, it might be more suitable to speak of a BGB, as there is abundant evidence that this barrier can be different from the BNB or can at least function differently (23–25). Other reasons for the BGB being incomplete can be postulated and include its embryological origin [i.e., the trigeminal ganglion is derived from neural crest cells and the trigeminal placode of the neural tube (26, 27)] and its neuroendocrine function [e.g., neuropeptides are released from and enter the trigeminal ganglion (28, 29)].

**Figure 6:**
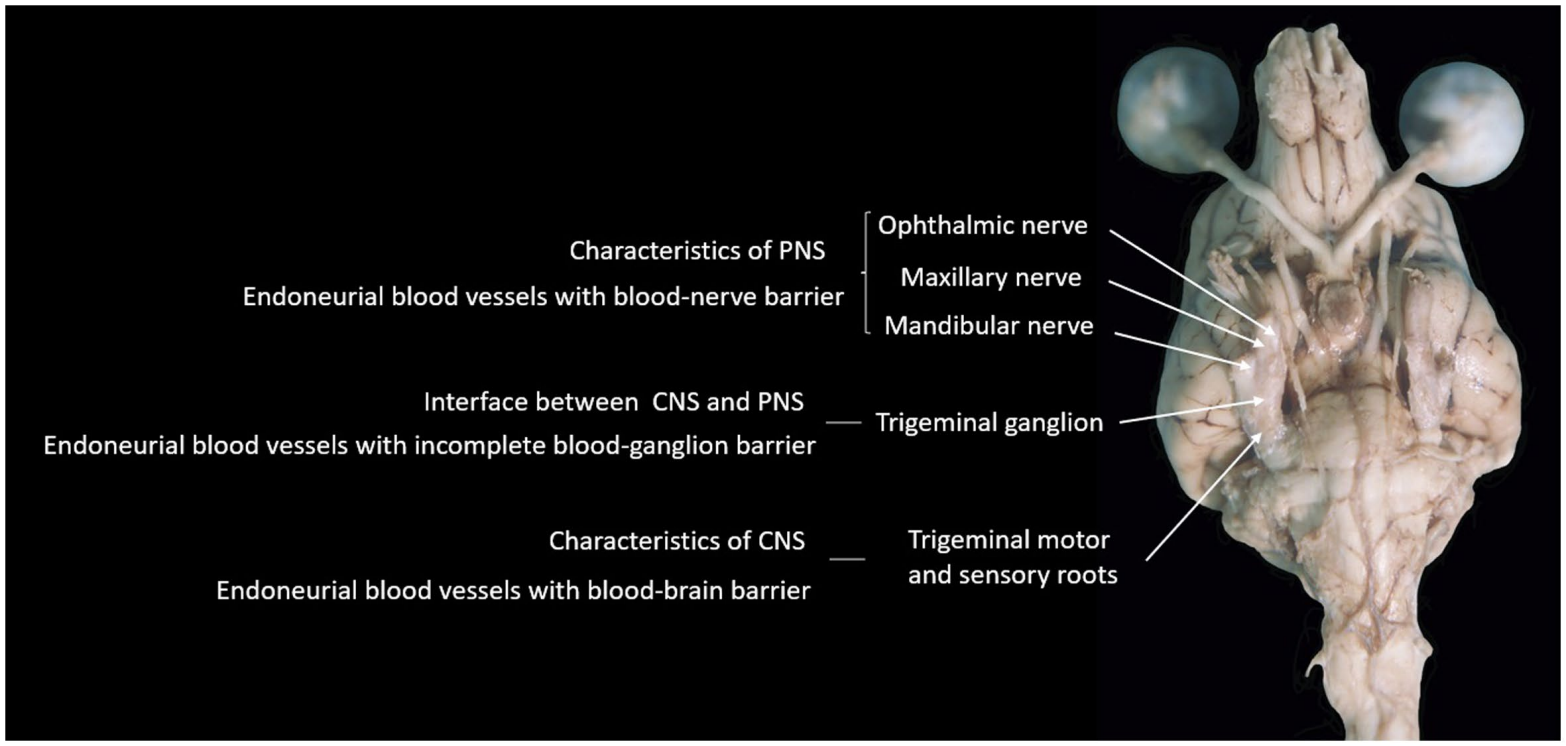
Photograph of the ventral aspect of a formalin-fixed encephalon of a dog, summarizing the hypothesis supported by findings in this study.

Whatever the reasons may be, the fact that the BGB is incomplete is evidenced and supported by the finding that the trigeminal ganglion is not the only ganglion reported to show contrast enhancement on MRI in humans. Indeed, other ganglia reported to show contrast enhancement on MRI in humans include lumbar spinal DRG (30), geniculate ganglion of the facial nerve (31), and superior cervical ganglion (32). The authors are not aware of specific reports on the contrast-enhancing features of such structures in dogs. Figure 6 summarizes our hypothesis regarding the incomplete BGB of the trigeminal ganglion with relevance to TGCE. The relative lack of reports specifically describing non-pathological contrast enhancement of other (cranial nerve) ganglia in dogs is likely related to the small size of these ganglia and difficulty in visualizing them in MRI studies in general, as well as in assessing contrast enhancement. In other words, the trigeminal ganglion is the largest and, therefore, TGCE was the most likely to have been noticed and reported by clinicians. Future studies looking into the presence or absence of non-pathological contrast enhancement of cranial nerve ganglia as well as DRG in dogs are warranted to elucidate this matter. In further support to the ‘incomplete BGB-theory’, studies reporting TGCE in dogs as a normal finding include images supporting true TGCE rather than mistakenly interpreting the presence of TGCE due to enhancement of periganglionic vascular structures (1, 2, 6, 7).

The clinical importance of TGCE as normal feature on MRI studies deserves to be stressed here. Diagnosis of pathological states of the trigeminal ganglion should not solely be based on contrast-enhancement, though it may well be that contrast-enhancement of, for example, trigeminal ganglionitis or neoplasia affecting the trigeminal ganglion is different from normal TGCE. However, there are no specific reports quantifying the extent of TGCE in pathological versus normal states in dogs. Reports documenting MRI-based diagnoses of trigeminal neuritis focus on contrast-enhancement as well as increased size (33). Reports documenting histopathologically confirmed cases of trigeminal ganglionitis with information on MRI appearance are rare. One report documented diffuse enlargement and contrast-enhancement of the trigeminal nerve (34). Some quantitative MRI data regarding size of the canine trigeminal nerve are published (35, 36). Future studies comparing healthy versus affected trigeminal nerves and ganglia may provide more information on the effects of certain pathological states on size and contrast-enhancement thereof.

Our findings of leptomeninges and a subarachnoid space (trigeminal cistern – a term derived from human literature (37) but not included in the Nomina Anatomica Veterinaria) around the trigeminal nerve roots and trigeminal ganglion mirror those reported in humans (38, 39). It is reported that the point of transition from meningeal to epi-perineurial covering is variable in people (39). Future studies including more specimens would be needed to verify individual variability of this transition in dogs.

The trigeminal cave [(cavum trigeminale or ‘Merckel cave’ – a term derived from human literature (38–41) but not included in the Nomina Anatomica Veterinaria] in dogs was subtle in the specimens examined in this study. Others have not named this anatomical feature ‘trigeminal cave’ in dogs, as the anatomy differs from that in humans (14). Use of this term may have some merit to facilitate discussion of pathology affecting this location. However, we do recognize that the term does not refer to the exact same anatomical features across species, as there are notable differences as has been previously described (14). Human studies have reported asymmetry of the Merckel Cave within individuals and variable morphology between individuals (39). This is likely to be true for dogs as well, especially when considering different breeds. Various pathologic processes have been described at this anatomical site in humans, affecting structures contained within and the associated with the bony lining itself (41). Future studies would be needed to document on the variability in these dimensions among individuals of the same breed or between different breeds of dogs.

Limitations to this study include the small number of canine specimens included and, in particular, the lack of electron microscopy to further support current hypotheses included in our discussion. Future studies including electron microscopy to evaluate for the presence or absence of, e.g., fenestration, tight junctions or other junctional complexes in capillaries in the trigeminal ganglion will be of vital importance to support the ‘incomplete BGB-theory’ as an explanation for TGCE in dogs. Immunohistochemistry studies on a well-fixed non-decalcified tissue could also provide evidence about the presence or absence of tight-junctions and BGB organization. However, various possible markers for such studies are not validated for canine tissues. Finally, future MRI studies to evaluate for the presence or absence of TGCE and compare it to enhancement of the complex vascular network surrounding it would be useful. In particular, specific sequences might be incorporated in those studies, such as a contrast enhanced 3D CISS sequence (15). Together with the results of our current study, such studies could provide veterinary neurologists and radiologists with valuable information to compare with imaging results of patients.

In conclusion, this study provides further support to the theory that TGCE in dogs may be due an incomplete BNB or BGB at the interface between the CNS with an intact BBB and leptomeninges and the PNS with a BNB and endo-, peri-, and epineurium rich in vessels.

## Data availability statement

The original contributions presented in the study are included in the article/Supplementary material, further inquiries can be directed to the corresponding author.

## Ethics statement

Ethical approval was not required for the studies involving animals in accordance with the local legislation and institutional requirements because the dogs were euthanized for medical reasons unrelated to the central nervous system (CNS). Written informed consent was not obtained from the owners for the participation of their animals in this study because they were donated by owners following the approved donation program of the University and used for anatomical dissections.

## Author contributions

KS: Conceptualization, Formal analysis, Writing – original draft, Writing – review & editing. EG: Conceptualization, Formal analysis, Writing – review & editing. MP: Conceptualization, Formal analysis, Investigation, Methodology, Writing – review & editing. VA: Visualization, Writing – review & editing, Conceptualization, Formal analysis, Investigation, Methodology.

## Funding

The author(s) declare financial support was received for the research, authorship, and/or publication of this article. The publication fee was covered by IVC Evidensia’s fund for publication of peer-reviewed scientific articles.

## Conflict of interest

The authors declare that the research was conducted in the absence of any commercial or financial relationships that could be construed as a potential conflict of interest.

## Publisher’s note

All claims expressed in this article are solely those of the authors and do not necessarily represent those of their affiliated organizations, or those of the publisher, the editors and the reviewers. Any product that may be evaluated in this article, or claim that may be made by its manufacturer, is not guaranteed or endorsed by the publisher.

## Supplementary material

SUPPLEMENTARY FIGURE 1 Schematic drawing of a nerve with associated blood vessels.

SUPPLEMENTARY VIDEOS 1-2 VAG provides two videos showing parts of the dissection process and microanatomy discussion.
