# Supplementary material for: Microanatomical findings with relevance to trigeminal ganglion enhancement on post-contrast T1-weighted magnetic resonance images in dogs
Source: Front Vet Sci. 2023 Sep 13;10:1256947. doi: 10.3389/fvets.2023.1256947 (PMC10533922; doi:10.3389/fvets.2023.1256947)
Supplement: SUPPLEMENTARY FIGURE 1 — Schematic drawing of a nerve with associated blood vessels. [file Data_Sheet_1.PDF]

Supplementary Material

Microanatomical Findings with Relevance to Trigeminal Ganglion Enhancement on Post-Contrast T1-Weighted Magnetic Resonance Images in Dogs

Koen M. Santifort[1,2*], Eric N. Glass[3], Marti Pumarola[4], Vicente Aige Gil[5]

[1]IVC Evidensia Small Animal Referral Hospital Arnhem, Neurology, Arnhem, The Netherlands

[2]IVC Evidensia Small Animal Referral Hospital Arnhem, Neurology, Arnhem, The Netherlands

[3]Red Bank Veterinary Hospital, Section of Neurology and Neurosurgery, Tinton Falls, United States of America

[4]Unit of Compared and Murine Pathology, Department of Animal Medicine and Surgery, Faculty of Veterinary Medicine, Universitat Autònoma de Barcelona, Campus UAB, Barcelona, Spain

[5]Department of Sanitat i Anatomía Animal, Faculty of Veterinary Medicine, Universitat Autònoma de Barcelona Campus UAB, Barcelona, Spain

*** Correspondence:**
K.M. Santifort
koen.santifort@evidensia.nl

Supplementary figure 1
Schematic drawing of a nerve with associated blood vessels. 1: Vasa nervorum and branching epineurial vessels (lymphatic vessels not shown), 2: epineurium, 3: interface between epineurial, perineurial and endoneurial vessels, 4: perineurium, 5: endoneurium, 6: nerve fascicle, 7: axon, 8: endoneurial vessels, 9: endothelial cell, 10: tight junction, 11: pericyte or satellite cell.

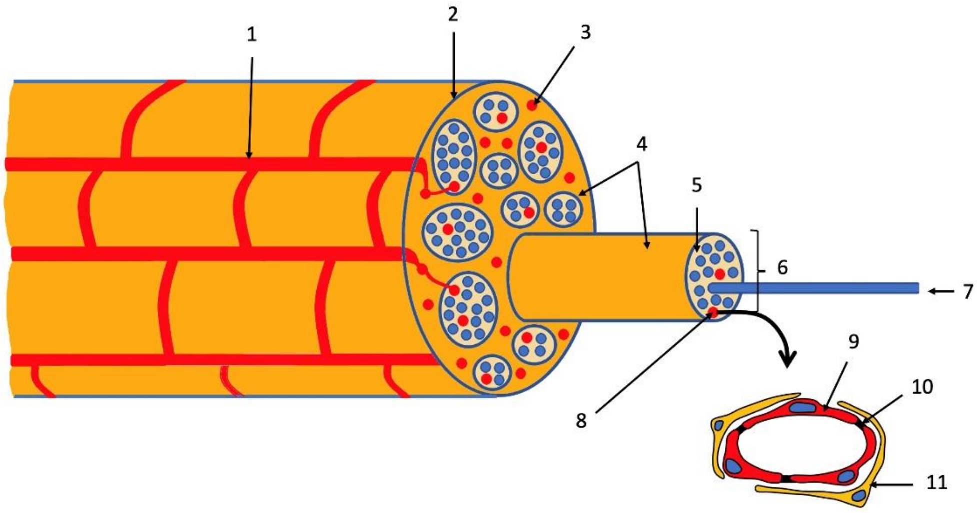

Supplementary videos

One of the authors (VAG) provides two videos showing parts of the dissection process and microanatomy discussion on his website: https://www.neuroanatomyofthedog.com/ and freely available on YouTube at https://www.youtube.com/watch?v=8Fg38KAoETk and https://www.youtube.com/watch?v=FtStg9oxX24&t.
